# Prevalence of Depression, Anxiety and Stress among Adult Population: Results of Yazd Health Study

**Published:** 2019-04

**Authors:** Mohsen Mirzaei, Seyed Mojtaba Yasini Ardekani, Masoud Mirzaei, Ali Dehghani

**Affiliations:** 1 Yazd Cardiovascular Research Center, Shahid Sadoughi University of Medical Sciences, Yazd, Iran.; 2 Department of Psychiatry, Research Center of Addiction and Behavioral Sciences, Shahid Sadoughi University of Medical Sciences, Yazd, Iran.; 3 Department of Epidemiology and Biostatistics, School of Public Health, Shahid Sadoughi University of Medical Sciences and Health Services, Yazd, Iran.

**Keywords:** *Anxiety*, *Anxiety and Stress Scale (DASS)*, *Depression*, *Iran*, *Prevalence*, *Stress*

## Abstract

**Objective:** The burden of mental disorders continues to grow with significant impacts on health. This study aimed to investigate the prevalence of stress, anxiety, and depression in Yazd adult population and to examine the associated socioeconomic factors.

**Method** **:** In a 2-step cluster sampling process, 10 000 residents of Yazd Greater Area (200 clusters of 50) were selected during 2014-2015. The short version of Depression, Anxiety and Stress Scale (DASS) Questionnaire was used to assess relevant depression, anxiety, and stress. Data were analyzed by chi-square test. All statistical analyses were done using SPSS version 16.0. P-values less than 0.05 were considered statistically significant.

**Results: **Depression, anxiety, and stress were seen in 29%, 32.2%, and 34.8% of adult residents of Yazd Greater Area, respectively. The symptoms of the disorders were moderate, severe, and very severe in 18.2%, 20.2%, and 23.4% of the population, respectively. Also, a significant difference was found between the symptoms of depression, anxiety, and stress and sex, age group, education, employment, marriage status, and country of birth. Of the total population under study, 3.7% were depressed, 7.7% were anxious, 9.5% had stress alone and 16.4% had symptoms of all the 3 disorders. Frequency of depression among Zoroastrians was more than muslims (42.1% vs 29.7%).

**Conclusion: **Despite achievemments in higher education and economic development of Yazd population over the past 2 decades, the trend of these disorders has alarmingly been increased. Considering the findings, it is necessary to develop evidence-based and appropriate community-based primary and secondary mental health prevention programs.

Mental health is one of the most important health indicators that causes considerable morbidity (1). According to the World Health Organization (WHO) report in 2008, one every five adults experienced mental disorders in the past year and 29.2% had a history of mental illness during their lifetime (2). To improve mental health, WHO has developed an evidence-based mental health program for the years 2013-2020 (3). 

Depression is a mood disorder which is characterized by short-term emotional responses to a serious health condition associated with impaired daily functioning accompanied by symptoms, such as sadness and frustration, feelings of guilt, insensibility, and loss of interest (4). 

Depression is a common psychiatric disorder in the world, affecting more than 300 million people worldwide (5, 6). Anxiety disorders are defined as a group of mental disorders characterized by an unpleasant feeling with uneasiness or worry about future events or the fear of responding to current events. It may occur without an identifiable triggering stimulus (4). In 2013, one out of every nine people in the world had at least one of the anxiety disorders (7, 8). In stress, a person's lack of compliance with environmental conditions leads to psychological and biological changes, and the person is at risk of becoming ill (9). 

Nearly a quarter of adults in the United States have psychiatric disorders, and almost half of them experience at least one mental illness during their lives (10). A systematic review and meta-analysis for global prevalence showed that the countries of Eastern Asia show an estimated one-year/lifetime prevalence less than other areas. The prevalence of one year among sub-Saharan African countries is low, while the highest lifetime prevalence was reported in Anglo-Celtic countries (11). 

The prevalence of having mental disorder over the past 12 months among Iranian population aged 18-64 years in 2011 was 23.6%, followed by anxiety disorders (15.6%). Two thirds of the patients had moderate to severe mental disorders (12). Recent findings indicated an increase in the prevalence of psychiatric disorders between 1999 and 2015 in Iranian adults (2). There is a variation in the prevalence of mental disorders in different Iranian provinces and across various groups from 11.7% to 38.9%. It is necessary to conduct further studies on the general population and use validated inventories to monitor changes in the future (13, 14). 

Several studies reported the prevalence of mental disorders in Yazd province. However, estimations from previous studies demonstrated inconsistency in the prevalence of mental disorders, especially depression, reporting the highest prevalence of depression (54.3%) in Iran (15) and the lowest level of happiness among the Iranian provinces (16). These differences may be due to non-representative sampling or using different inventories. Given that a large population-based study on psychiatric disorders has not been conducted in Yazd until now, such study was needed. Most similar studies were conducted on small groups of Yazd population, such as diabetic patients (17), college students (18), and truck drivers (19). 

This study aimed to investigate the prevalence and associated predictors of stress, anxiety, and depression in Yazd adult population using data from a large population-based study and associated predictors. 

Comparison of findings with previous population-based studies may help health system managers to design and implement appropriate interventional strategies for health promotion.

## Materials and Methods

Yazd Health Study (YaHS) is a prospective study conducted to determine the prevalence of non-communicable diseases and associated risk factors in Yazd Greater Area. Details of the methodology were published elsewhere (20). 

In a nutshell, the population frame of YaHS was adults aged 20-69 years living in Yazd Greater Area. In a 2-step cluster sampling process, 10 000 participants (200 clusters of 50) were selected during 2014-2015. Data were collected using a questionnaire. DASS (Depression, Anxiety and Stress Scale) Questionnaire (short form) was used, which is a different, simple, and approved instrument for assessing depression, anxiety, and stress both in clinical settings and in the community (21, 22). DASS is a short screening tool that measures depression, anxiety, and stress by a 21-item self-report questionnaire. For each disorder, seven questions are considered, and the final score is obtained by the total score of the questions related to it. Each question was scored using a Likert scale, ranging from 0 (did not apply to me at all/never) to three (applied to me very much, or most of the time/almost always). Higher scores indicated a higher level of disorder by a specific classification scoring. Individuals are classified as normal, mild, moderate, severe, and very severe based on their responses. Various studies have shown high concurrent validity for Beck Depression Inventory, Beck Anxiety Inventory, and DASS. Also, this tool can detect stress symptoms from depression and anxiety (23). Comparing the results from DASS-21 with the diagnosis of psychiatric interviews showed the sensitivity and specificity of 75% to 89% for this tool and its potential for accurate screening of depression, anxiety, and stress (24, 25). The reliability and validity of the translated version of the Persian questionnaire was confirmed for an Iranian population (26, 27). Sahebi et al reported a Cronbach’s alpha of 0.77, 0.79, and 0.78 for depression, anxiety, and stress subscales, respectively. The correlation coefficient of this inventory was 0.7 with Beck’s Inventory, 0.67 with Zung Anxiety Test, and 0.49 with Perceived Stress Inventory (26). 

For direct age standardization of the prevalence rates, Yazd population details were obtained from the Statistical Center of Iran. Categorical variables were presented as frequencies and percentages. The prevalence of depression, anxiety, and stress was described as proportions. Chi square test was used for categorical variables to analyze the differences in demographic variables between the groups. All statistical analyses were done using SPSS version 16. P-values less than 0.05 were considered statistically significant.

This study was approved by Ethics Committee of Shahid Sadoughi University of Medical Sciences (No. 17/1/73941). Informed consent was obtained from all the participants.

## Results

**Figure 1:**
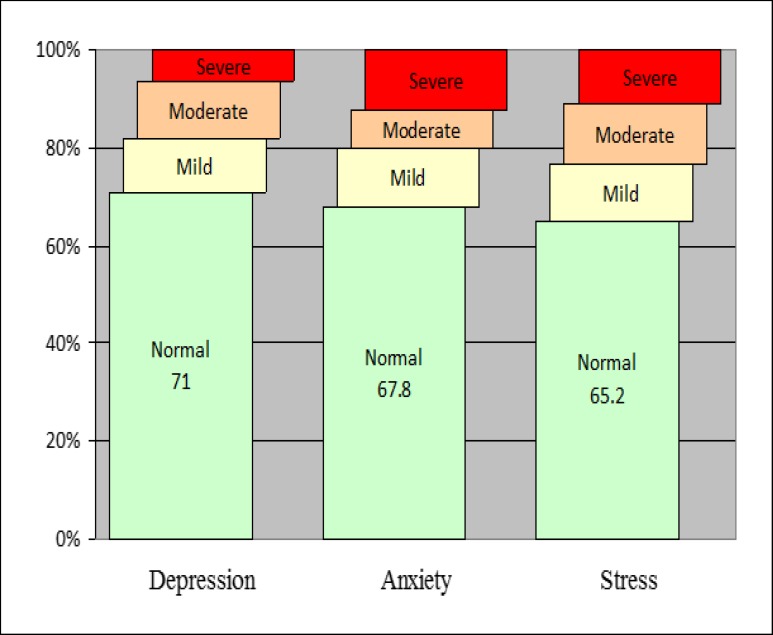
Prevalence of Age-Standardized Depression, Anxiety, and Stress in Adults Aged 20-69 Years Living in Yazd, Iran, During 2014–2015

A total of 9965 people participated in this study. Depression, anxiety, and stress were found in 29%, 32.2%, and 34.8% of adult residents of Yazd Greater Area. The symptoms of the disorders were moderate to very severe in 18.2%, 20.2%, and 23.4% of the population, respectively (Figure 1). 

**Figure 2:**
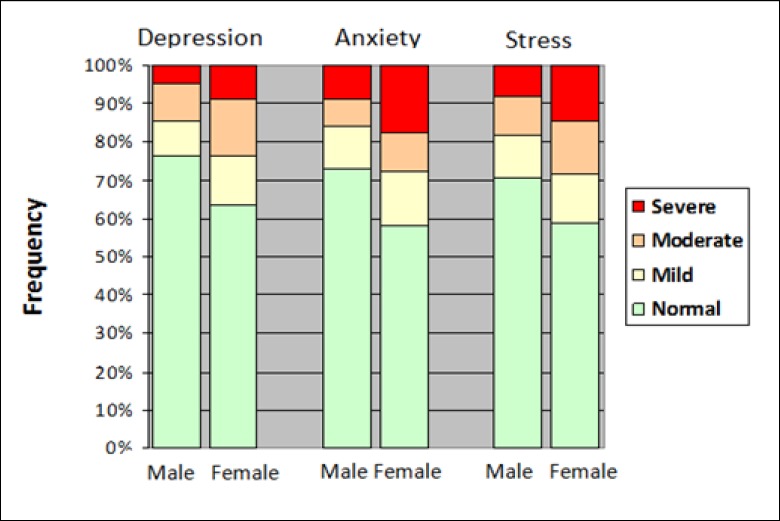
Prevalence of Age-Standardized Depression, Anxiety, and Stress by Sex in Adults Aged 20-69 Years Living in Yazd, Iran, During 2014–2015

Figure 2 demonstrates that the prevalence of depressive symptoms was significantly higher in women than in men (36.5 vs 23.7%). 

The prevalence and severity of symptoms in older adults were significantly higher than the younger population. There was a significant difference between the symptoms of depression and education, employment, marriage status, and country of birth. The prevalence was higher in less educated, unemployed, uninsured, and immigrants. Frequency of depression among Zoroastrians was more than Muslims (42.1 vs 29.7%) (Table 1). Also, 28.9% of the population had a history of depression, which was higher in people with severe depressive symptoms compared to the rest. Table 1 demonstrates the prevalence and severity of depressive symptoms in adolescents according to demographic variables.

Table 2 shows that 32.2% of the adults had symptoms of anxiety, which was more in women compared to men (41.9 vs 36.7%, P-value <0.0001) (Figure 2). As age increased and education decreased, the prevalence and severity of anxiety significantly increased. Employed, insured, and those with Iranian nationality were less anxious than others (P-value <0.0001). Widowed and divorced women had the highest frequency of anxiety and single people had the lowest level. 

There was no significant difference in the frequency of anxiety symptoms among Muslims and Zoroastrians.

Women had 11.8% more stress than men. Moderate to severe intensity of stress was also significantly higher in women (Table 3). Younger adults aged 20-29 years were more under stress than other age groups, and stress significantly decreased with increase in years of education.

There was less stress in the employed and insured people. Immigrants both from other provinces of Iran and other countries were more likely to be stressed compared to non-migrant Yazd residents (P-value = 0.003). 

**Figure 3:**
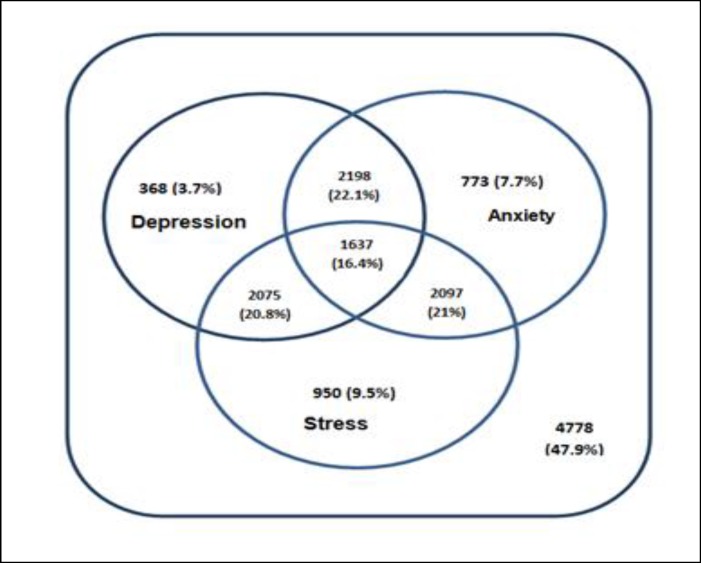
Overlap of Depression, Anxiety, and Stress in Adults Aged 20-69 Years Living in Yazd, Iran, During 2014–2015

Of the participants, 47.9% did not have any symptoms of depression, anxiety, and stress. Moreover, 3.7% were depressed, 7.7 were anxious, 9.5% had stress, and 16.4% had symptoms of all the 3 disorders. Figure 3 shows the coincidence of the presence of symptoms of depression, anxiety, and stress in individuals.

**Table 1:** Prevalence of Depression According to Various Factors in Yazd Adult Population Aged 20-69 Years during 2014–2015 (n = 9965)

**Subgroups**	**Normal**	**Depression**			**P value**
		**Mild**	**Moderate**	**Severe & Very Severe**	
Crude	6926 (69.9%)	1098 (11.1%)	1208 (12.2%)	681 (6.9%)	
Age group					
20-29	1449 (73.8%)	195 (9.9%)	201 (10.2%)	118 (6%)	<0.0001
30-39	1416 (69.9%)	224 (11.1%)	247 (12.2%)	138 (6.8%)	
40-49	1411 (68.9%)	242 (11.8%)	265 (12.9%)	131 (6.4%)	
50-59	1378 (70%)	198 (10.1%)	230 (11.7%)	163 (8.3%)	
60-69	1272 (66.7%)	239 (12.5%)	265 (13.9%)	131 (6.9%)	
Education					
Primary school and less	1635 (63.2%)	325 (12.6%)	387 (15%)	240 (9.3%)	<0.0001
High school	1871 (66.8%)	340 (12.1%)	380 (13.6%)	211 (7.5%)	
Diploma& Graduate diploma	2181 (74.4%)	274 (9.3%)	311 (10.6%)	166 (5.7%)	
BSc	1005 (77.8%)	120 (9.3%)	108 (8.4%)	58 (4.5%)	
MSc. and Doctorate	197 (77.6%)	28 (11%)	22 (8.7%)	7 (2.8%)	
History of depression					
Yes	169 (28.9%)	99 (16.9%)	149 (25.5%)	168 (28.7%)	<0.0001
No	6678 (72.8%)	974 (10.6%)	1038 (11.3%)	489 (5.3%)	
Employment					
Employed	2994 (76.3%)	360 (9.2%)	389 (9.9%)	183 (4.7%)	<0.0001
Unemployed	2371 (62%)	493 (12.9%)	586 (15.3%)	375 (9.8%)	
Housewife	1482 (72.7%)	221 (10.8%)	220 (10.8%)	116 (5.7%)	
Insurance					
Not insured	326 (60.7%)	66 (12.3%)	83 (15.5%)	62 (11.5%)	<0.0001
Insured	6536 (70.6%)	1008 (10.9%)	1102 (11.9%)	615 (6.6%)	
Migration status					
Native	5245 (70.4%)	813 (10.9%)	894 (12%)	496 (6.7%)	<0.001
From within the province	654 (69.9%)	110 (11.8%)	123 (13.1%)	49 (5.2%)	
From other provinces	855 (67.6%)	145 (11.5%)	156 (12.3%)	109 (8.6%)	
From overseas	130 (60.5%)	23 (10.7%)	35 (16.3%)	27 (12.6%)	
Marriage status					
Married	6002 (71.2%)	902 (10.7%)	990 (11.7%)	536 (6.4%)	<0.0001
Single	727 (69%)	118 (11.2%)	125 (11.9%)	84 (8%)	
Widowed	178 (46.8%)	68 (17.9%)	79 (20.8%)	55 (14.5%)	
Divorced	21 (38.2%)	9 (16.4%)	14 (25.5%)	11 (20%)	
Country of birth					
Iranian	6754 (70%)	1068 (11.1%)	1173 (12.2%)	654 (6.8%)	<0.001
Non-Iranian	130 (60.5%)	23 (10.7%)	35 (16.3%)	27 (12.6%)	
Religion					
Muslim	6696 (70.3%)	1034 (10.8%)	1148 (12%)	653 (6.9%)	<0.0001
Zoroastrian	136 (57.9%)	41 (17.4%)	42 (17.9%)	16 (6.8%)	

**Table 2:** Prevalence of Anxiety According to Various Factors in Yazd Adult Population Aged 20-69 Years during 2014–2015 (n = 9965)

**Subgroups**	**Normal**	**Anxiety**			**P value**
		**Mild**	**Moderate**	**Severe & Very Severe**	
Crude	6508 (65.7%)	1239 (12.5%)	852 (8.6%)	1314 (13.3%)	
Age group					
20-29	1406 (71.6%)	217 (11.1%)	131 (6.7%)	209 (10.6%)	<0.0001
30-39	1382 (68.2%)	245 (12.1%)	140 (6.9%)	258 (12.7%)	
40-49	1343 (65.5%)	249 (12.2%)	186 (9.1%)	271 (13.2%)	
50-59	1239 (62.9%)	272 (13.8%)	171 (8.7%)	287 (14.6%)	
60-69	1138 (59.7%)	256 (13.4%)	224 (11.7%)	289 (15.2%)	
Education					
Primary school and less	1464 (56.6%)	354 (13.7%)	288 (11.1%)	481 (18.6%)	<0.0001
High school	1756 (62.7%)	371 (13.2%)	261 (9.3%)	414 (14.8%)	
Diploma, Graduate Diploma	2080 (70.9%)	337 (11.5%)	216 (7.4%)	299 (10.2%)	
BSc	978 (75.8%)	145 (11.2%)	65 (5%)	103 (8%)	
MSc. and Doctorate	189 (74.4%)	33 (13%)	16 (6.3%)	16 (6.3%)	
History of depression					
Yes	163 (27.9%)	91 (15.6%)	89 (15.2%)	242 (41.4%)	<0.0001
No	6247 (68.4%)	1139 (12.4%)	740 (8.1%)	1026 (11.2%)	
Employment					
Employed	2892 (73.7%)	414 (10.5%)	249 (6.3%)	371 (9.4%)	<0.0001
Unemployed	2146 (56.1%)	558 (14.6%)	413 (10.8%)	708 (18.5%)	
Housewife	1396 (68.5%)	251 (12.3%)	174 (8.5%)	218 (10.7%)	
Insurance					
Not insured	322 (60%)	64 (11.9%)	46 (8.6%)	105 (19.6%)	<0.0001
Insured	6130 (66.2%)	1157 (12.5%)	786 (8.5%)	1188 (12.8%)	
Migration status					
Native	4929 (66.2%)	925 (12.4%)	632 (8.5%)	962 (12.9%)	<0.0001
From within the province	611 (65.3%)	126 (13.5%)	96 (10.3%)	103 (11%)	
From other provinces	812 (64.2%)	163 (12.9%)	103 (8.1%)	187 (14.8%)	
From overseas	112 (52.1%)	24 (11.2%)	20 (9.3%)	59 (27.4%)	
Marriage status					
Married	5569 (66.1%)	1081 (12.8%)	701 (8.3%)	1079 (12.8%)	<0.0001
Single	752 (71.3%)	105 (10%)	74 (7%)	123 (11.7%)	
Widowed	162 (42.6%)	51 (13.4%)	68 (17.9%)	99 (26.1%)	
Divorced	22 (40%)	6 (10.9%)	7 (12.7%)	20 (36.4%)	
Country of birth					
Iranian	6352 (65.8%)	1214 (12.6%)	831 (8.6%)	1252 (13%)	<0.0001
Non-Iranian	112 (52.1%)	24 (11.2%)	20 (9.3%)	59 (27.4%)	
Religion					
Muslim	6264 (65.7%)	1226 (12.6%)	839 (8.6%)	1288 (13.2%)	<0.0001
Zoroastrian	149 (63.4%)	30 (12.8%)	21 (8.9%)	35 (14.9%)	

**Table 3:** Prevalence of Stress According to Various Factors in Yazd Adult Population Aged 20-69 Years during 2014–2015 (n = 9965)

**Subgroups**	**Normal**	**Stress**			**P value**
		**Mild**	**Moderate**	**Severe & Very Severe**	
Crude	6445 (65%)	1149 (11.6%)	1205 (12.2%)	1114 (11.2%)	
Age group					
20-29	1317(67.1%)	215 (11%)	241 (12.3%)	190 (9.7%)	0.033
30-39	1278 (63.1%)	241 (11.9%)	267 (13.2%)	239 (11.8%)	
40-49	1310 (63.9%)	254 (12.4%)	267 (13%)	218 (10.6%)	
50-59	1281 (65.1%)	214 (10.9%)	223 (11.3%)	251 (12.7%)	
60-69	1259 (66%)	225 (11.8%)	207 (10.9%)	216 (11.3%)	
Education					
Primary school and less	1492 (57.7%)	310 (12%)	378 (14.6%)	407 (15.7%)	<0.0001
High school	1813 (64.7%)	340 (12.1%)	326 (11.6%)	323 (11.5%)	
Diploma& Graduate Diploma	2003 (68.3%)	329 (11.2%)	332 (11.3%)	268 (9.1%)	
BSc	934 (72.3%)	125 (9.7%)	137 (10.6%)	95 (7.4%)	
MSc. and Doctorate	176 (69.3%)	35 (13.8%)	26 (10.2%)	17 (6.7%)	
History of depression					
Yes	188 (32.1%)	76 (13%)	108 (18.5%)	213 (36.4%)	<0.0001
No	6174 (67.3%)	1051 (11.5%)	1074 (11.7%)	880 (9.6%)	
Employment					
Employed	2737 (69.7%)	458 (11.7%)	408 (10.4%)	323 (8.2%)	<0.0001
Unemployed	2329 (60.9%)	436 (11.4%)	502 (13.1%)	558 (14.6%)	
Housewife	1300 (63.8%)	236 (11.6%)	280 (13.7%)	223 (10.9%)	
Insurance					
Not insured	326 (60.7%)	66 (12.3%)	83 (15.5%)	62 (11.5%)	<0.0001
Insured	6536 (70.6%)	1008 (10.9%)	1102 (11.9%)	615 (6.6%)	
Migration status					
Native	4984 (66.9%)	823 (11%)	853 (11.5%)	788 (10.6%)	<0.0001
From within the province	530 (56.6%)	129 (13.8%)	156 (16.7%)	121 (12.9%)	
From other provinces	769 (60.8%)	166 (13.1%)	168 (13.3%)	162 (12.8%)	
From overseas	119 (55.3%)	28 (13%)	27 (12.6%)	41 (19.1%)	
Marriage status					
Married	5557 (65.9%)	969 (11.5%)	1001 (11.9%)	903 (10.7%)	<0.0001
Single	687 (65.2%)	117 (11.1%)	131 (12.4%)	119 (11.3%)	
Widowed	186 (48.9%)	52 (13.7%)	55 (14.5%)	87 (22.9%)	
Divorced	21 (38.2%)	8 (14.5%)	17 (30.9%)	9 (16.4%)	
Country of birth					
Iranian	6283 (65.1%)	1118 (11.6%)	1177 (12.2%)	1071 (11.1%)	0.002
Non-Iranian	119 (55.3%)	28 (13%)	27 (12.6%)	41 (19.1%)	
Religion					
Muslim	6214 (65.2%)	1097 (11.5%)	1141 (12%)	1079 (11.3%)	0.003
Zoroastrian	135 (57.4%)	31 (13.2%)	46 (19.6%)	23 (9.8%)	

## Discussion

The prevalence of mental disorders has increased in Yazd population over the last two decades (1, 28). Approximately, half of Yazd population (52.1%) had a mild to severe major depression, anxiety, and stress, and 16.4% had symptoms of all the 3 disorders. 

Three National Mental Health Surveys in Iran in 2001, 2002, and 2012 reported a prevalence of psychiatric disorders of 21.0%, 17.1%, and 23.6%, respectively (1, 12, 14). The difference in the results could be due to using different inventories, including Beck, Schizophrenia and Affective Disorders Scale (SADS), Composite International Diagnostic Interview (CIDI), and General Health Questionnaire (GHQ), different range of psychiatric disorders and age range/sample size of the studies. However, this increase may be true to some extent, particularly because the prevalence of these psychiatric disorders in Yazd population is alarmingly high compared to the Iranian national average.

Our findings are similar to those of three studies in Tehran, which reported a prevalence of psychiatric disorders of 21.5%, 34.3%, and 39.7%, respectively, in the years 2000, 2009, and 2012 using the GHQ-28 questionnaire (28-30). This can be due to the similarity of the GHQ-28 inventory in measuring anxiety and depression in target groups. High prevalence of mental disorders in Yazd, similar to a cosmopolitan city such as Tehran, is not expected and more studies should be conducted on the role of socioeconomic factors, such as urbanization, industrialization, and migration. 

Ahmadi et al reported that the prevalence of psychiatric sorders in Yazd children and adolescents was as high as 35.5% (anxiety, 22.2%) in 2016, which is consistent with our findings and justifies the high prevalence in adults as well (31). With the lack of community-based preventive intervention programs for younger age groups, the high incidence of mental disorders in adults is expected.

The increasing trend of psychiatric disorders in adulthood and high prevalence of these disorders in childhood mandate a comprehensive prevention programs and life skills training to reduce the incidence of mental disorders in Yazd adult population(31). In 2015, 23.4% of Iranian adult populations were suspected of having at least 1 mental disorder, and the prevalence of anxiety and somatization symptoms was higher than social dysfunction and depression symptoms (2). Considering that psychiatric disorders consisted of 4 alignments in this study, it can be said that prevalence of depression (29%), anxiety (32.3), and stress (34.8%) is reasonably high in Yazd Greater Area. 

Our finding showed that the prevalence of psychiatric disorders was significantly higher in women than in men in Yazd. Depression (36.5% vs 23.7%), anxiety (41.9% vs 26.7%), and stress (40.9% vs 29.1%) were all higher in women than in men in Yaz. Similarly, according to a study in Iran (2011), using CIDI and MDQ inventories, it was found that 26.5% of women and 20.8% of men had psychiatric disorders, anxiety disorder (19.4% vs 12%), and mood disorders (17.3 vs 11.9) (12). 

In all the mentioned three national studies, results were indicative of higher prevalence of psychiatric disorders in women compared to men (27.6% vs 14.9%; 27.9% vs 28.6%; 42.4% vs 36.4%), which is consistant with findings of this study (28). Other studies reported that common mental disorders are more common in women than in men in other countries (32, 33). This difference can be attributed to socioeconomic disadvantage, cultural constraints, and violence. Women are more likely to express mental health problems than men. Evidence suggests the role of sex hormones in such gender differences (34). 

This study showed that the prevalence and severity of depression was significantly higher in older adults, immigrants, less educated, unemployed, and uninsured people. These findings are in line with the study by Mohammadi et al in which psychiatric disorder is more prevalent in adults aged 41-55 years compared to other adult age groups.(1). 

There was an association between place of birth and depression, anxiety, and stress symptoms in this study. Those born in other provinces and countries were more depressed, anxious, and stressful than those who were born in Yazd, similar to other studies (35, 36). The higher prevalence of depression in immigrants and religious minorities was also similar to that of other studies, which may be due to the differences in language, culture, and pattern of receiving health care (37). 

 This study revealed that anxiety was more common in women, those with higher income, less educated, unemployed, uninsured, widowed, and divorced indviduals. These findings are consistent with the reults of the Noorbala et al study that showed the risk of mental disorder increases with age, illiteracy/lower education, divorce, widownesss, unemployment, and being chronically ill (2, 38). 

Findings of this study are consistent with the results of numerous studies on the relationship between socioeconomic status, marital status (39, 40), older age (41), lower education (42), unemployment (43) and higher prevalence of psychiatric disorders. Biological changes and disabilities in older age, economic problems in the unemployed, inability to control social problems in people with less education, and social constraints in divorced people may explain the higher rate of psychiatric disorders in these groups, similar to the findings of other studies conducted in Iran (2) and in the world (11). These social determinants of health were identified as psychosocial risk factors that increase mental disorders. However, more studies are needed to confirm the association between these factors and mental disorders.

## Limitation

This study had several limitations. In this study, only the symptoms of depression, anxiety, and stress were examined, and other important psychiatric disorders, such as personality disorders, were not reported. Like most large population-based studies, a validated questionnaire was filled by trained interviewers to assess depression, anxiety, and stress. Thus, this cross-sectional analysis may not confirm cause and effect relationships.

However, the main strength of this study was population representativeness. This study investigated common mental disorders and relevant predictors. Therefore, this study might have underestimated the overall mental disorders and provided a conservative result.

## Conclusion

Over the past 2 decades, the trend of depression, anxiety, and stress has been increased in Yazd Greater Area. This demands more root-cause analysis and ethiologic and interventional studies to investigate the causes of this high prevalence and to prevent and control these disorders. More studies are needed to evaluate the contribution of probable factors, such as daily stress, social media, interpersonal skills, and economic problems.
